# Optimizing the Infusion Route of Human Bone Marrow Mesenchymal Stromal Cells to Mitigate Liver Ischemia–Reperfusion Injury in a Porcine Model

**DOI:** 10.3390/cells14191496

**Published:** 2025-09-24

**Authors:** Stefan H. Luijmes, Job P. van Kooten, Henk P. Roest, Jubi de Haan, Michail Doukas, Cornelia J. Verhoeven, Kairong Wang, Jorke Willemse, Luc J. W. van der Laan, Monique M. A. Verstegen, Jeroen de Jonge

**Affiliations:** 1 Erasmus MC Transplant Institute, Department of Surgery, Division of Hepatopancreatobiliary and Transplant Surgery, University Medical Center Rotterdam, 3015 GD Rotterdam, The Netherlands; 2 Department of Intensive Care Medicine, Erasmus University Medical Center, Dr. Molewaterplein 40, 3015 GD Rotterdam, The Netherlands; 3 Department of Pathology, Erasmus University Medical Center, Dr. Molewaterplein 40, 3015 GD Rotterdam, The Netherlands

**Keywords:** ischemia–reperfusion injury, mesenchymal stromal cells, regenerative medicine

## Abstract

Mesenchymal stromal cells (MSC) have been shown to mitigate IRI through their anti-inflammatory and immune-modulating capacities. This study aims to demonstrate the feasibility, safety, and effectiveness of hepatic administration of bone marrow-derived (BM)-MSCs in a large pig model relevant to human anatomy. After complete vascular exclusion for 45 min, 3 × 10^6^ human BM-MSCs/kg body weight were infused via the portal vein or hepatic artery. BM-MSC infusion did not cause obstruction of hepatic or pulmonary blood flow within 6 h after infusion. Cells were effectively retained in the liver, being undetectable in peripheral blood, lung, and spleen samples. Human B2M expression, as a marker for BM-MSC presence, was significantly higher for the left liver lobe in arterial infusion compared to portal infusion. In liver samples with high BM-MSC levels, we identified the prevention of up- or downregulation of some genes related to inflammation and energy metabolism that was present in non-treated control samples, indicating biological effects within 6 h of infusion. We conclude that hepatic BM-MSC infusion is feasible and safe, with the hepatic artery serving as the optimal administration route for homogenous distribution. These findings pave the way for clinical studies on MSC infusion in IRI, either in situ in liver conditions or ex situ during machine perfusion.

## 1. Introduction

Ischemia–reperfusion injury (IRI) is defined as the exacerbation of cellular injury and death following restoration of blood supply to ischemic tissues [1]. IRI is a common cause of post-surgical liver injury, including resection and transplantation. During (warm) ischemia, the disruption in blood flow leads to the depletion of adenosine triphosphate, mitochondrial dysfunction, and the switch from aerobic to anaerobic metabolism [2]. Subsequent reperfusion exacerbates tissue damage via the formation of reactive oxygen species and the production of pro-inflammatory cytokines, such as interleukin-1 and tumor necrosis factor-alpha [3,4]. This elicits a sterile inflammation response through the activation of both innate and adaptive immune systems, which consequently leads to cellular dysfunction [4,5]. Current treatment for IRI relies on supportive measures, but cell-based therapies may offer an alternative to attenuate the severity of IRI and accelerate liver regeneration.

Mesenchymal stromal cells (MSCs) are multipotent cells that have the potential to repair injured tissue by interacting with immune cells through direct cell-to-cell interaction or the secretion of paracrine factors, leading to immunomodulation [6,7]. The use of a directly available allogeneic, “off the shelf” MSC product in patients presenting with IRI seems to be a promising strategy to improve surgical and transplant outcomes following reperfusion.

Injecting MSCs for therapeutic applications requires efficient migration and homing to the target site. Previous studies have shown that systemically administrated MSCs accumulate in the lungs and have a short survival time [8,9,10]. Despite the chemoattractant capabilities of MSCs to migrate to injured tissue sites [11,12], there is no evidence that systemically infused MSCs will effectively migrate to the liver [13,14,15]. By direct infusion of MSCs in the liver, a higher local dose can be delivered, potentially enhancing the therapeutic effect due to the avoidance of rapid clearance in the systemic circulation. In cases of open liver surgery (i.e., transplantation or extended resection), direct administration of MSCs in the hepatic circulation is feasible. In other circumstances, infusion via the portal vein or hepatic artery cannulation is possible through interventional radiology techniques.

Although several studies on the application of MSCs in IRI have been conducted in mouse and rodent models [16,17,18,19], the potential of MSCs in the treatment of IRI in large animals has not yet been assessed. Additionally, the optimal route of direct intrahepatic administration and the safety of delivering large numbers of MSCs directly into the different vascular beds of the liver have not yet been studied. We therefore performed a study in pigs using an ischemia–reperfusion injury model to induce hepatic damage, after which human bone marrow-derived MSCs (BM-MSC) were infused either in the portal vein or hepatic artery. The primary outcome measure was safety, defined as the preservation of hepatic vascular flow, absent detection of BM-MSCs in hepatic venous drainage, and the absence of pulmonary hypertension. Secondary outcomes were defined as the distribution of BM-MSCs across the liver and proof of biological effects on the liver tissue.

## 2. Materials and Methods

### 2.1. Ethics and Animals

Purpose-bred, female Yorkshire pigs (n = 16) were kept at the animal facility of the Erasmus University Medical Centre (Rotterdam, The Netherlands) one week prior to the experiment. During this period, animals had free access to food and water till twelve hours before the experiment, when food was replaced with glucose-enriched water. All animal experiments were performed with permission from the animal ethics commission (DEC) of the Erasmus University Medical Centre (DEC protocol number: 105-14-05).

### 2.2. In Vitro Expansion and Characterization of BM-MSC

Cryopreserved human BM-MSCs (Fr90 BM-MSC M0121 and BM13; Neostem, New York, NY, USA) [20] were thawed by stepwise dilution and expanded (maximum of 6 passages) in culture medium consisting of alpha-minimum essential medium (MEM)/GLUTAMAX (Gibco, Paisley, UK), 2% fetal bovine serum (Sigma-Aldrich, St. Louis, MO, USA), 1% antibiotic–antimycotic (Gibco) supplemented with 20 ng/mL of recombinant human epidermal growth factor (eBioscience, San Diego, CA, USA), and 10 ng/mL of recombinant human fibroblast growth factor 2 (eBioscience) at 37 °C with 5% CO_2_. BM-MSCs were harvested on the day of the experiment and were consequently characterized by flow cytometry. Staining for surface markers of BM-MSCs was performed with allophycocyanin (APC), labeled anti-human CD90, R-phycoerythrin (PE)-conjugated anti-human CD105, and fluorescein isothiocyanate (FITC)-conjugated human CD73 (Becton Dickinson, San Jose, CA, USA). Samples were analyzed on a FACS Canto II flow cytometer (BD Biosciences, Vianen, The Netherlands) using FlowJo software (version 10.6.1, Treestar, Ashland, OR, USA).

### 2.3. Experimental Procedure

**Figure 1:**
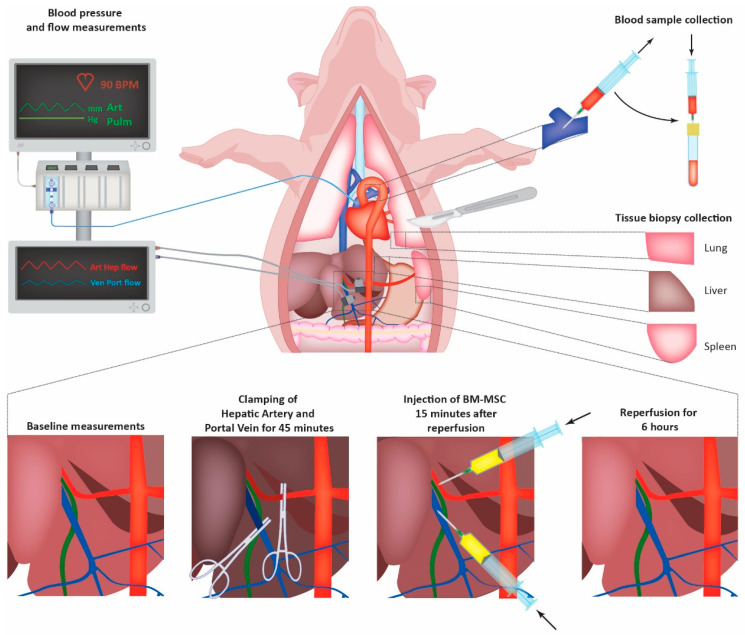
A schematic overview of experimental liver ischemia and reperfusion and BM-MSC treatment in pigs. A tracheostomy was performed to insert a Schwann–Ganz catheter for the measurement of pulmonary artery pressure (PAP). After midline laparotomy, the hepatic artery and portal vein were found for the placement of flow probes for continuous blood flow measurements. At baseline, biopsies (liver, lung, spleen) were taken, and hepatic blood flow, PAP, and general hemodynamics were measured, after which the portal vein, hepatic artery, and hepatic veins were clamped for 45 min. Following reperfusion, either no BM-MSCs or 3 × 10^6^ BM-MSCs/kg were infused via the hepatic artery or portal vein. During follow-up, the previously mentioned hemodynamics were measured, and blood samples of the pulmonary artery were taken to detect BM-MSCs in circulation. Six hours after reperfusion, biopsies of the liver, lung, and spleen were taken, and the experiment was terminated. Abbreviations: BM-MSCs, bone marrow-derived mesenchymal stromal cells.

Animals were sedated using 30 mg/kg ketamine (Alfasan, Woerden, The Netherlands) and 1 mg/kg Midazolam (Actavis, Hafnarfjordur, Iceland) intramuscularly, after which they were transported to the animal operating theatre where they were further anesthetized using Midazolam (1.5 mg/kg/h), ketamine (5 mg/kg/h), and Sufentanil (Sufentaforte, Janssen-Cilag, Tilburg; 4 mcg/kg/h). After induction of anesthesia, tracheotomy was performed for intubation and mechanical ventilation, after which a central venous line, central arterial line, and pulmonary arterial Swann–Ganz catheter (Edwards Life Sciences LLC, Irvine, CA, USA) were inserted. Pigs underwent laparotomy via midline incision, after which the hepatoduodenal ligament was identified and dissected to expose the proper hepatic artery and portal vein. Flow probes (PS series T206 flow meter, Transonic, Elsloo, The Netherlands) were placed around the proper hepatic artery and portal vein, continuously measuring hepatic blood flow. In parallel, BM-MSCs were harvested and characterized, after which they were put in 5 mL of phosphate-buffered saline (Lonza, Basel, Switzerland) before infusion. Details about the expansion and characterization of BM-MSCs are listed in the supplementary data. The liver was totally excluded of blood flow for forty-five minutes by placing vascular-type clamps on the proper hepatic artery, portal vein, and hepatic veins (Figure 1). After forty-five minutes, blood flow was restored by removing the clamps. Fifteen minutes after reperfusion, 3 × 10^6^ BM-MSCs/kg bodyweight were infused directly in the hepatic artery through catheterization of the gastroduodenal artery or directly injected through a needle in the portal vein. The number of infused MSCs per kg bodyweight was based on previous pig studies in the field of acute liver failure [21,22]. Hemodynamics and hepatic blood flow were continuously monitored and recorded during the entire experiment (SC 9000XL, Siemens Medical Systems Inc., Danvers, MA, USA). After six hours of reperfusion, pigs were euthanized by infusing 10 mL of 100 mg/mL potassium chloride (B Braun, Melsungen, Germany) into the central venous line.

### 2.4. Tissue and Blood Sampling

Biopsies of the right and left liver lobe (peripheral and central), right and left lung (upper and lower zone), and spleen were collected prior to vascular exclusion and 6 h after reperfusion. Lung, liver, and spleen biopsies were either fixed in 4% formalin or stored in RNA-later (Qiagen, Venlo, The Netherlands) for histological and transcriptomic analysis, respectively. Arterial blood samples were acquired prior to vascular exclusion and after reperfusion at several timepoints. Arterial blood samples were obtained prior to vascular exclusion (0 min) and 30, 60, 90, 120, 180, 240, 300, and 360 min after reperfusion. Blood samples were collected in BD SST vacutainers (Becton Dickinson, Breda, The Netherlands) and centrifuged (18 min, 1300 g, room temperature) to obtain serum. The levels of aspartate transferase (AST), alanine transaminase (ALT), alkaline phosphatase (AF), and gamma-glutamyl transferase (GGT) were detected with a biochemical analyzer (Cobas 8000, Diagnostics, Basel, Switzerland).

### 2.5. RNA Extraction, cDNA Preparation, and RT-qPCR

Tissue samples (spleen, lung, liver) were submerged in Trizol Cell Lysis Reagent (Qiagen) and homogenized using glass beads. RNA was isolated with the MiRNeasy kit (Qiagen) according to manufacturer’s protocol using a standard chloroform RNA extraction protocol. RNA content was determined using photospectrometry (Nanodrop 2000, Thermo-Scientific, Waltham, MA, USA). RNA content was normalized before reverse transcriptase transcription. cDNA was made by adding 2 µL PCR reaction mix (PrimeScript RT master mix, Takara Bio, Japan) to 500 ng RNA, in a total of 10 µL dH_2_O, in a 2720 thermal cycler (Applied Biosystems, Waltham, MA, USA). RT-qPCR was performed with the primers that are listed in Table S1. Each reaction mixture contained 5 µL cDNA template, 10 µL SYBR Select PCR Master mix (Applied Biosystems, Life technologies, Warrington, UK), and 2 µL primers in a total reaction volume of 20 µL. qPCR was performed in a StepONE Plus Real-time PCR machine (Applied Biosystems), according to the manufacturer’s guidelines, using the following scheme: 95 °C for 2 min, 40 cycles of 95 °C for 15 s, followed by 72 °C for 1 min. The value of porcine glyceraldehyde-3-phosphate dehydrogenase (GAPDH) was taken as the housekeeping gene for determining the dCt of human B2M expression. A cycle threshold (CT) of 40 was taken in cases of an undetermined threshold. All RT-qPCR data are presented as 2^(-dCT)^.

### 2.6. Bulk RNA Sequencing

RNA from liver tissue was extracted using the NucleoSpin RNA Kit (Macherey-Nagel, Düren, Germany) following the manufacturer’s instructions. For each individual sample, messenger RNA was purified from total RNA using poly-T oligo-attached magnetic beads. After fragmentation, first-strand cDNA was synthesized using random hexamer primers, followed by second-strand cDNA synthesis using dTTP for a non-directional library. Libraries were sequenced using the Ilumina NovaSeq X Plus platform (Novogene Ltd., Cambridge, UK). Reads with adapter contamination, over 10% of uncertain nucleotides, or with >50% low-quality nucleotides (Base quality < 5) were removed, which resulted in 60–120 million, 150-nucleotide long, paired-end clean reads. The resulting datasets were mapped against the Ensembl 110 Sus scrofa genome (assembly 11.1) using HISAT2, and mapped reads were converted to counts using FeatureCounts with the Sus scrofa genome assembly 11.1 associated annotation file (Ensembl Sus scrofa 11.1.110.gtf.gz). Raw count files were uploaded to the Galaxy Web platform public server at usegalaxy.org for downstream analysis [23]. Differentially expressed genes were identified using DESeq2 for the following pairwise factors: ‘Baseline’, ‘Ischemic Control’, ‘Arterial Infusion’, and ‘Portal Infusion’. Transcripts with an adjusted *p*-value < 0.05 (Benjamin Hochberg FDR corrected) were considered differentially expressed for each comparison. Gene set enrichment analysis (GSEA) was performed using the UCSD/Broad institute package for GSEA [24]. Three publicly available datasets with regard to ischemia–reperfusion data were obtained from EMBL-EBI Ontology (IRI set 1, https://www.ebi.ac.uk/ols4/ontologies/efo/classes?short_form=EFO_0000556, assessed on 24 October 2024), Harmonizome 3.0 (IRI set 2, https://maayanlab.cloud/Harmonizome/gene_set/ischemiareperfusion/GeneRIF+Biological+Term+Annotations, assessed on 21 October 2024), and GEO database (IRI set 3, Wang et al. [25]).

Raw data are available from the GEO repository at NCBI (accession number GSE282013) upon reasonable request.

### 2.7. Tracking BM-MSC in Pulmonary Circulation

To assess the presence of BM-MSCs in the pulmonary circulation, blood samples (1 mL) from the pulmonary artery were collected at 1, 3, 5, and 10 min after injection of BM-MSCs. A sample before BM-MSC infusion (baseline) was taken as the negative control. Tracking of the BM-MSCs was performed via staining with allophycocyanin (APC), labeled anti-human CD90, R-phycoerythrin (PE)-conjugated anti-human CD105, and fluorescein isothiocyanate (FITC)-conjugated human CD73 (Becton Dickinson, San Jose, CA, USA). Samples were analyzed on a FACS Canto II flow cytometer (BD Biosciences, Vianen, The Netherlands) using FlowJo software (version 10.6.1, Treestar, Ashland, OR, USA).

### 2.8. Immunohistochemistry

Formalin-fixed liver biopsies were embedded in paraffin and 4 µm sections were prepared for hematoxylin and eosin (HE) staining according to standard protocols. The slides were analyzed by a liver pathologist, blinded to the context of the cases.

### 2.9. Statistical Analyses

Baseline characteristics were described as the median with 25–75% percentiles (interquartile range, IQR) and were analyzed with the Kruskal–Wallis test. The results were expressed as the mean ± standard deviation (SD). The Mann–Whitney test or unpaired *t*-test was used for intergroup analysis. For continuous repeated variables, a mixed model (two-way ANOVA) was used with Sidak’s post hoc test for multiple comparisons. Data were analyzed using SPSS (software version 24, IBM corp., Armonk, NY, USA), Labchart (version 7, ADInstruments, Colorado Springs, CO, USA), and Graphpad (version 6.0, Graphpad Software Inc., La Jolla, CA, USA). A two-sided significance level of 0.05 was chosen.

## 3. Results

### 3.1. Establishment of the Hepatic Ischemia–Reperfusion Injury Model

**Figure 2:**
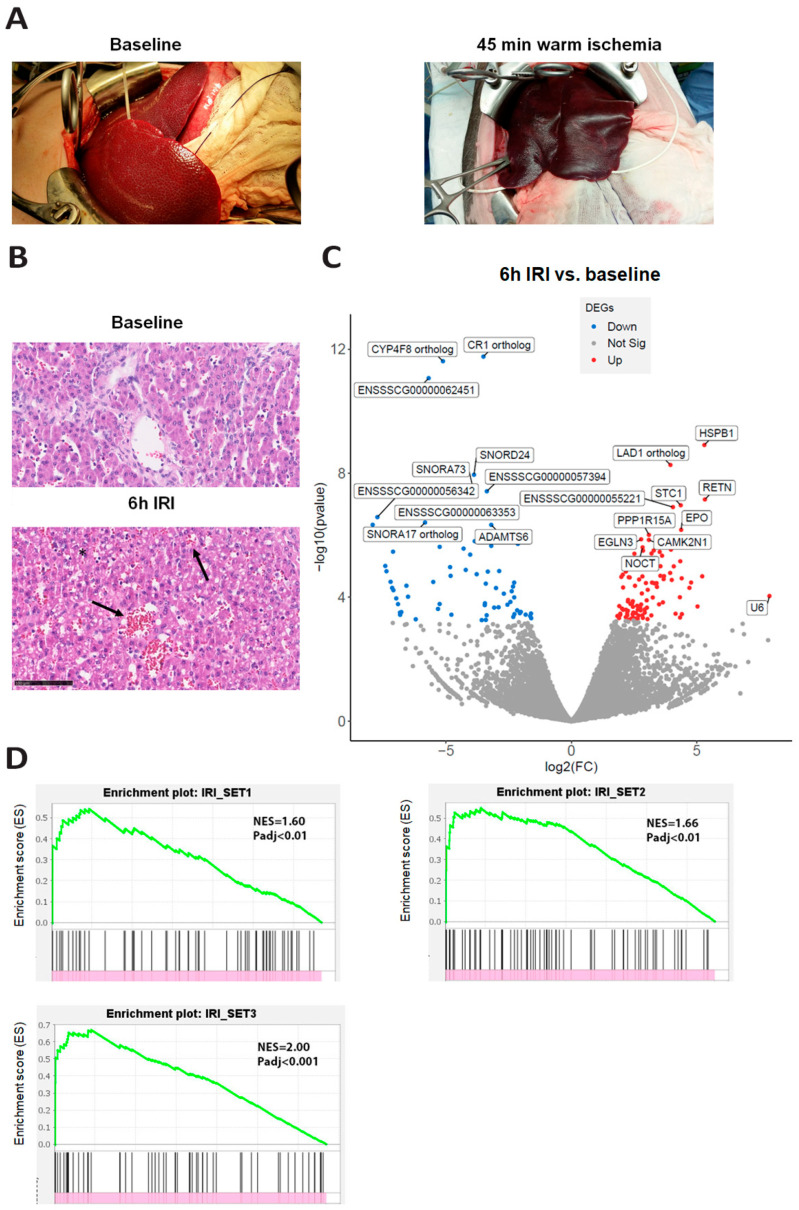
The effects of ischemia–reperfusion injury on macroscopic, histological, and transcriptional levels. (**A**) A macroscopic view of a porcine liver at baseline (left) and after 45 min of warm ischemia (right). (**B**) A representative image of hematoxylin and eosin staining in liver tissue at baseline (left) and at 6h reperfusion (right). The lower panel shows the presence of congestion (arrows) and hepatocellular vacuolization (asteryx) in the hepatic lobule after 6 h of reperfusion. Scale bar indicate 100 µm. (**C**) Differentially expressed gene expression analysis based on RNA bulk sequencing of baseline versus IRI at 6 h reperfusion. The volcano plot shows significantly up and downregulated genes at baseline compared to IRI (false discovery rate, FDR < 0.05). Each dot represents a single gene, with genes that are significantly upregulated in red, genes that are significantly downregulated in blue, and non-significant genes in gray. (**D**) Gene set enrichment analysis (GSEA) of the up-and downregulated genes at baseline compared to IRI versus three publicly available gene sets related to ischemia and reperfusion. IRI set 1: EMBL-EBI Ontology (top left panel, *p* < 0.01); set 2: Harmonizome 3.0 (top right panel, *p* < 0.01); and set 3: Wang et al. [25] (bottom left panel, *p* < 0.001). The x-axis shows the ranked list of differentially expressed genes and the vertical bars show genes that belong to the matched gene set. The y-axis shows the enrichment score (ES) that represents the degree to which genes are (statistically) over-represented (or under-represented) in the ranked dataset. The green line connects the points of ES and genes.

Clamping of the hepatic vasculature and consequent restoration of the circulation resulted in profound macroscopic changes (Figure 2A). Sinusoidal congestion and vacuolization of the hepatocellular cytoplasm were seen on the HE staining at 6 h of reperfusion (Figure 2B). Transcriptomic analysis showed that 170 genes were differentially expressed at the end of IRI (Figure 2C). These differentially expressed genes included genes related to hypoxia (EPO and CXCR4) and inflammation (GZMB), which were upregulated. Among the downregulated genes were genes involved in synthetic function and hepatic metabolism (CYP4F8, PPARA, CYP7A1). No gene sets with hallmark pathways were significantly enriched at 6 h of reperfusion compared to baseline (Figure S1). However, gene set enrichment analysis showed a significant overlapping gene signature of our gene set with publicly available IRI-related datasets (Figure 2D).

Serum levels of aspartate transferase (AST) and alanine transaminase (ALT), alkaline phosphatase (AF), and gamma-glutamyl transferase (GGT) showed an increase after reperfusion in all groups (Figure S2). There were no significant differences in liver and biliary injury levels between the treatment groups and control group at any timepoint after reperfusion.

### 3.2. Characterization of BM-MSC and Hemodynamic Effects

BM-MSCs were plastic-adherent, elongated, and had a spindle-shaped appearance (Figure S3A). Flow cytometry showed that BM-MSCs expressed CD73, CD90, and CD105 and were negative for CD34, CD133, CD45, and CD44 (Figure S3B–D and S4). Cell numbers injected per kilogram bodyweight were equal between treated groups (*p* = 0.45, Figure S3E), as was animal body weight (Table 1). Considering hemodynamic and respiratory values, there were no significant differences between experimental groups in heart rate, mean arterial blood pressure, mean pulmonary arterial blood pressure, blood oxygen saturation, end tidal CO_2_, and temperature (Table 1).

**Figure 3:**
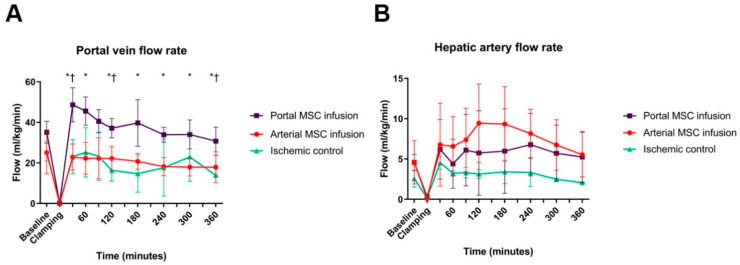
Portal MSC infusion led to vasodilation in the portal vein, whereas this effect was not seen in the arterial MSC infusion group. (**A**) A rebound in portal vein flow was observed in all groups after releasing the clamps on the hepatic vasculature. Increased blood flows (mL/kg/min) were measured in the groups that received BM-MSCs via the portal vein compared to the control group and the group that received cells via the hepatic artery. The difference in portal vein flow between the portal MSC infusion and ischemic control groups reached statistical significance at 120 min after reperfusion. There was a significant difference in portal flow between the portal MSC infusion and arterial MSC groups at 30 and 240 min after reperfusion. † *p* < 0.05 portal group vs. control group. * *p* < 0.05 portal group vs. arterial group. (**B**) Hepatic artery flow was restored to the values that were measured at baseline. No significant differences in hepatic artery blood flow (mL/kg/min) were measured between the portal MSC infusion group, arterial MSC infusion group, and ischemic control group.

After unclamping, blood flow rebounded to the baseline level in the hepatic artery and portal vein (Figure 3A,B). Interestingly, after BM-MSC infusion in the portal vein, portal venous flow persistently increased compared to ischemic controls and the arterial injected group. This difference between the portal BM-MSC infusion and ischemic control groups was statistically significant at 120 min after reperfusion, and reached significance at 30 and 240 min compared with arterially infused BM-MSCs (Figure 3A). Although apparently the same phenomenon was observed in arterial blood flow with arterially injected BM-MSCs, these differences were not significant between treated groups and the control group (Figure 3B).

Two adverse events occurred not related to BM-MSC infusion. One pig in the experimental group receiving the BM-MSCs via the portal vein presented with a pulmonary air embolism. This was most likely caused by wrongful flushing of the Swan–Ganz catheter. This experiment was prematurely stopped after unsuccessful resuscitation of the pig. After gene expression analysis by qPCR on lung tissue biopsies, no detectable expression of B2M was found, suggesting that this incident was not a thrombo-embolism caused by the MSCs. In the arterial MSC infusion group, we encountered one case of a non-correctable spasm or dissection of the hepatic artery. Most likely this was caused by traumatic overclamping of the artery. Gene expression analysis using qPCR showed no difference in human-specific B2M expression within the right and left liver lobe of this animal compared to other arterially injected subjects.

### 3.3. First-Pass Retention of BM-MSCs in Liver and No Localization to Circulation or Other Tissues

**Figure 4:**
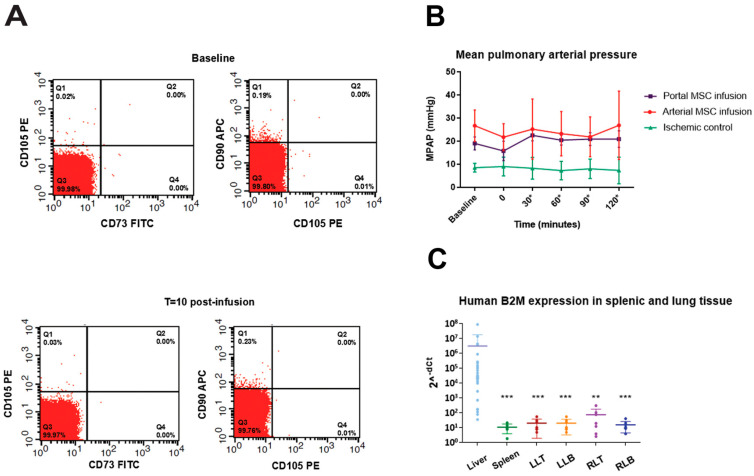
Human BM-MSCs infused in the liver were undetectable in the pulmonary circulation, did not affect pulmonary artery pressures, and were not detectable in lung and spleen tissue. (**A**) Flow cytometry analysis revealing the absence of human CD73, CD90, and CD105 markers in blood obtained from the pulmonary artery at baseline (top panels) and at 10 min after hepatic infusion (bottom panels). Also, at 1, 3, and 5 min, no cells positive for CD73, CD90, and CD105 were detected. The representative gating strategy is displayed in Figure S5. (**B**) Baseline mean pulmonary arterial pressures (MPAPs) were not different from the values that were measured in the first 120 min following BM-MSC infusion in the treated groups. * minutes after BM-MSC infusion. (**C**) No clear human B2M signal was detected in lung and spleen tissue samples compared to liver tissue at 6 h after reperfusion. A cycle threshold (CT) value of 40 was taken in cases of undetermined CT values during RT-qPCR. Gene expression of human B2M is displayed as 2^-dCt^. ** *p* < 0.001 vs. liver, *** *p* < 0.0001 vs. liver. Abbreviations: B2M, beta-2 microglobulin; LLB, left lung base; LLT, left lung top; RLB, right lung base; RLT, right lung top.

Flow cytometry analysis revealed no detectable levels of human CD90, CD105, and CD73-positive cells in the pulmonary artery samples at different timepoints post BM-MSC administration, demonstrating that cells did not localize to the pulmonary circulation (Figure 4A). No increase in pulmonary arterial pressure, in comparison to baseline pressure, was observed in the first 120 min post reperfusion in any of the treatment groups that were investigated, indicating that pulmonary resistance did not increase after injection of BM-MSCs. There was no difference in pulmonary arterial pressure between the arterial and portal MSC infusion group (Figure 4B). According to qPCR analyses on lung and spleen samples, no measurable levels of human B2M were detected (Figure 4C).

**Figure 5:**
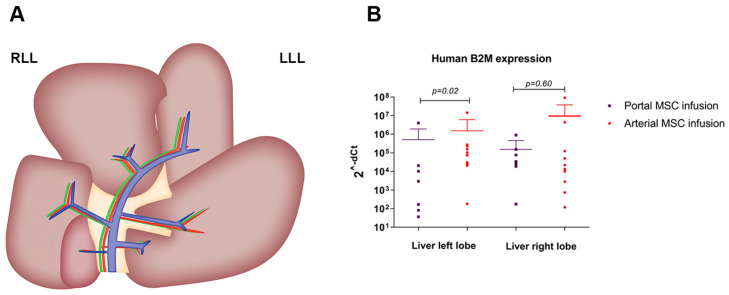
Arterial MSC infusion shows better delivery of BM-MSCs versus portal infusion for the left liver lobe. (**A**) porcine liver anatomy with a branching pattern of the portal vein and hepatic artery. Biopsies were obtained in the left liver lobe (LLL) and right liver lobe (RLL) for RNA extraction and consequent qPCR analysis. (**B**) Human beta-2-microglobulin (B2M) expression in the left liver lobe was significantly higher in the arterial MSC infusion group compared to the portal MSC infusion group (*p* = 0.02). This might be attributed to the rheology of portal blood with preference to the right lobe branches. There was no significant difference between the arterial group and the portal group regarding human B2M expression in the right liver lobe (*p* = 0.60). Gene expression of human B2M is displayed as 2^-dCt^.

Regarding the distribution of the BM-MSCs in the liver (Figure 5A), gene expression analysis showed that significantly less B2M was detected in the left lobe when cells were administered through the portal vein compared to when BM-MSCs were administered through the hepatic artery (*p* = 0.02) (Figure 5B). No significant differences in the distribution of BM-MSCs over the right lobe were found between treated groups (*p* = 0.60).

### 3.4. Biological Effects of BM-MSC on IRI

**Figure 6:**
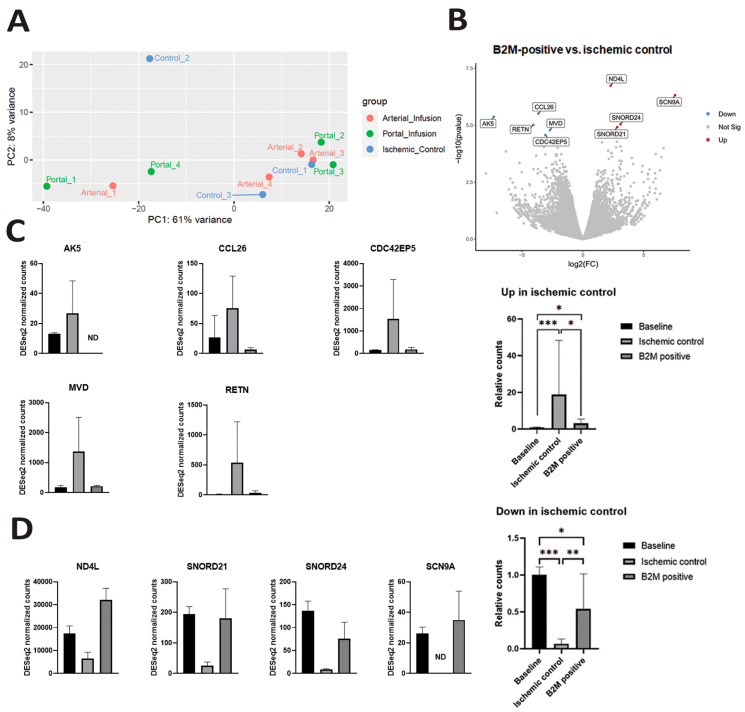
Transcriptomic analysis of porcine gene expression shows beneficial effect of BM-MSCs on hepatic IRI. (**A**) A principal component analysis (PCA) plot of RNA bulk sequencing data colored by groups (arterial infusion, portal infusion, ischemic control). Each data point represents an individual pig. (**B**) A volcano plot displaying the change in gene expression at 6 h after reperfusion for pig liver samples with relatively high human beta-2-microglobulin (B2M) levels compared to ischemic control samples. Each dot represents a single porcine gene, with genes that are significantly upregulated in red, genes that are significantly downregulated in blue, and non-significant genes in gray. In total, 9 differentially expressed genes (DEGs) were found (false discovery rate < 0.05). (**C**) The individual expression of genes that were upregulated in ischemic controls, including adenylate kinase 5 (AK5), C-C motif chemokine ligand 26 (CCL26), CDC42 effector protein 5 (CDC42EP5), mevalonate diphosphate decarboxylase (MVD, and retinin (RETN), were downregulated in B2M-positive samples at 6 h of reperfusion. Gene expression of the mentioned genes at baseline is displayed as a reference. Gene expression below the threshold of detection was characterized as non-detectable (ND). The error bar represents the mean ± SD. Count normalization of genes that were significantly upregulated after IRI compared to baseline and significantly downregulated in samples containing BM-MSCs (B2M-positive). A comparison between the groups was performed using the ANOVA test followed by the Kruskal–Wallis test. * *p* < 0.05; *** *p* < 0.001. (**D**) The individual expression of genes that were downregulated in ischemic controls and upregulated in B2M-positive samples at 6 h of reperfusion. The genes include NADH-ubiquinone oxidoreductase chain 4 (ND4), NADH-ubiquinone oxidoreductase chain 4L (ND4L), sodium voltage-gated channel alpha subunit 9A (SCN9A), small nucleolar RNA, C/D box 21 (SNORD21), small nucleolar RNA, and C/D box 24 (SNORD24) in the ischemic control group and B2M-positive samples. Gene expression of the mentioned genes at baseline is displayed as a reference. Gene expression below the threshold of detection was characterized as non-detectable (ND). The error bar represents the mean ± SD. Count normalization of genes that were significantly downregulated after IRI compared to baseline and significantly upregulated in the BM-MSC (B2M-positive) samples. A comparison between the groups was performed using the ANOVA test followed by the Kruskal–Wallis test. * *p* < 0.05, ** *p* < 0.01, and *** *p* < 0.001.

To examine the activation of infused BM-MSCs in response to hepatic IRI, a whole transcriptome comparison between samples collected after 6 h of follow-up (ischemic controls, arterial group, portal group) was performed (Figure 6A). Human B2M expression was assessed with qPCR within the analyzed sequencing samples. In total, nine differentially expressed genes were found between B2M-positive samples (containing BM-MSC) in comparison to ischemic controls at 6 h IRI (Figure 6B). Two inflammatory genes, C-C motif chemokine ligand 26 (CCL26) and resistin (RETN), were significantly upregulated after IRI in controls at 6 h reperfusion. This was almost completely prevented in the B2M-positive samples, although still significant differences with baseline were noticed (Figure 6C).

In addition, activation of three genes related to cell energy metabolism (adenylate kinase 5, AK5) and lipid synthesis (mevalonate diphosphate decarboxylase, MVD) was noticed in ischemic controls, which was significantly redressed by BM-MSCs.

An inversed pattern was found in four genes, downregulated in controls at 6 h reperfusion: NADH-ubiquinone oxidoreductase chain 4L (ND4L), small nucleolar RNA, C/D box (SNORD) 21, SNORD24, and sodium voltage-gated channel alpha subunit 9 (SCN9A) were significantly lower after IR in controls. ND4L is part of the mitochondrial genome and is involved in ATP synthesis. The role of SNORD21, SNORD24, and SCN9A in liver metabolism or inflammation is so far unknown, but recent research shows that snoRNAs play a pivotal role in proliferation, cell migration, apoptosis, and cell cycle progression in hepatocellular carcinoma (HCC) and related etiologies, such as hepatitis B virus (HBV), hepatitis C virus (HCV), and non-alcoholic fatty liver disease (NAFLD) [26]. In B2M-positive samples, this decrease was almost completely prevented, although differences compared with the baseline condition were still apparent (Figure 6D).

## 4. Discussion

IRI is an inevitable process during liver transplantation and hepatic surgery which leads to tissue injury and organ dysfunction. Reperfusion is essential to restore homeostasis following ischemia, but the re-establishment of blood circulation paradoxically leads to aggravation of the developed injury. Upon reperfusion, mitochondrial dysfunction causes a sterile inflammation response involving both the innate and adaptive immune systems [1,27]. By applying bulk RNA sequencing, we observed upregulation of hypoxia-related and inflammatory genes that are known to be involved in hepatic IRI. This suggests that inflammatory signals that are present in the microenvironment may activate MSCs, revealing their anti-inflammatory characteristics and enabling the prevention of excessive tissue damage the promotion of tissue repair by secreting cytokines such as IL-10 and other soluble factors [28]. Although MSCs can switch their pro- and anti-inflammatory role in response to inflammatory signals in the area, we here show that MSCs are activated towards an anti-inflammatory state, providing a promising approach to mitigating these IRI-related pathological processes.

In this study, we demonstrated the safety and feasibility of direct administration of BM-MSCs into the hepatic vascular beds in a porcine ischemia–reperfusion injury model. From a safety point of view, BM-MSCs did not migrate to the pulmonary circulation or spleen, and injection did not result in increased pulmonary artery pressure. The two adverse effects we encountered were attributed to technical issues and did not result from BM-MSC injection. We demonstrated that intrahepatic BM-MSC infusion of 3 million BM-MSCs per kilogram bodyweight (estimated about 10 million per 100 g of liver tissue) is well tolerated by the liver, and cells are contained in the hepatic vascular bed. However, our follow-up period was restricted to 6 h post infusion, which limits the monitoring of prolonged safety measures and survival. Future studies should be designed with longer follow-up times to assess the long-term safety outcomes of MSC therapy.

Several administration routes have been described for BM-MSC injection, including peripheral intravenous (IV) injection, intrasplenic injection, and direct administration via the portal vein or hepatic artery. Selection of the administration route may affect the number of engrafted BM-MSCs in the liver parenchyma, which in turn affects the biological and therapeutic effects. Peripheral intravenous administration was shown to be ineffective, with most cells ending up in the lung parenchyma and not reaching the site of injury [8,9,10].

Two groups showed benefit of direct intraportal administration over intravenous administration in a pig model of acute liver failure in terms of biological effects and outcomes [22,29]. In our current study, we demonstrate that BM-MSCs are more equally distributed over the different hepatic lobes when BM-MSCs are administered through the hepatic artery, compared to portal vein injection. Based on our findings and the literature, we therefore believe that arterial administration is superior to portal injection regarding cell distribution and the best option with regard to safety, feasibility, and therapeutic effects. During open liver surgery, MSCs can thus be injected through a catheter in the de gastroduodenal artery, not jeopardizing hepatic blood flow.

To explore the use of MSCs to mitigate IRI in liver transplantation, Laing et al. used normothermic machine perfusion to deliver BM-MSC-like multipotent adult progenitor cells in human discarded livers [30]. They demonstrated that intra-arterially delivered cells transmigrated across the vascular endothelium, which led to consistent engraftment in liver parenchyma, whereas portally injected cells tended to arrest within the sinusoidal channels. In a similar model, our group infused human labeled BM-MSCs during cold machine perfusion of porcine livers and consequently re-perfused the grafts [31]. There were no differences seen in cell distribution across the liver between arterial and portal vein injection, but increased cytokine production was observed after arterial infusion.

In cases of liver transplantation, we propose the application of MSCs to the arterial pole of machine perfusion to target IRI prior to implantation. A recent proof-of-concept study showed the potential to treat IRI without the use of living cells by incorporating an MSC-based bioreactor during NMP [32]. Liver-related inflammatory factors led to priming and consequent pro-regenerative secretory activity of MSCs, resulting in improved graft viability and cell energy status of porcine livers. The recent technological developments to perfuse donor livers for multiple days on the machine allow for treating injured donor livers with regenerative therapies (i.e., MSC), making them suitable for transplantation [33,34]. MSCs are currently manufactured to a clinical grade as allogenic “off the shelf” therapy, paving the way for clinical applications. For this, MSCs are typically expanded in serum-free culture media and with a chemically defined formulation that is designed to comply with good manufacturing practice (GMP) standards [35]. However, here we used culture medium that included fetal bovine serum and non-GMP growth factors for the expansion of BM-MSCs, which limits the clinical translatability.

The purpose of our study was to demonstrate the safety and feasibility of direct MSC-infusion for the treatment of IRI, and therefore, the experiment was ended after 6 h of reperfusion. Although we had a relatively short follow-up time, we found nine differentially expressed genes in samples that were positive for human B2M at 6 h of reperfusion. We observed downregulation of pro-inflammatory genes (CCL 26 and RETN) and upregulation of a gene related to cell energy metabolism (ND4L). We only looked at transcriptomic differences between groups after 6 h post infusion. Future studies should extend follow-up times to investigate gene expression changes over time following infusion. However, transcriptomic profiling may not capture the functional effects of MSC therapy as it lacks information about cellular signaling and protein activity. A multi-omics approach by integrating proteomics and metabolomics alongside transcriptomics might result in a better understanding of the functional (long-term) effects of MSC therapy in IRI. Also, we propose to investigate the effects of direct hepatic MSC infusion on renal tissue following IRI. Acute kidney injury is a major complication after hepatic IRI, resulting in comorbidity following transplantation or surgery [36,37,38]. Tautenhahn et al. demonstrated that acute kidney injury after extended liver resection was ameliorated by the paracrine activity of IV-infused BM-MSCs [39]. MSC treatment resulted in hemodynamic stabilization, improved intra-renal blood flow, and stimulated kidney repair. This suggests that, beyond the liver, the kidney may also benefit from the secretory activity of infused MSCs, thereby enhancing therapeutic efficacy.

## 5. Conclusions

In conclusion, this study shows that clinically relevant amounts of BM-MSCs can be safely administered directly into the liver vascular beds, without compromising the hepatic blood flow or causing cell migration to the lungs or spleen. We showed that arterial administration of BM-MSCs is superior to portal injection with regard to homogenous cell distribution over the liver lobes. On a transcriptomic level, the presence of BM-MSCs led to downregulation of pro-inflammatory genes and upregulation of a gene involved in cell energy metabolism in liver tissue within 6 h of administration, underlining a swift biological response to the presence of BM-MSCs. Our results pave the way for future studies in which the therapeutic long-term effects of MSC treatment on IRI—either in situ in liver conditions or ex situ during machine perfusion -can be further elucidated before possible progression to clinical application.

## Figures and Tables

**Table 1:** Animal characteristics and perioperative data. Abbreviations: bpm, beats per minute; IQR, interquartile range; HR, heart rate; MABP, mean arterial blood pressure; mm Hg, millimeters of mercury; MPABP, mean pulmonary arterial blood pressure; kg, kilogram; SpO_2_, blood oxygen saturation; etCO_2_, end tidal CO_2_. * Kruskal–Wallis test.

	Ischemic Control, n = 5 Median (IQR)	Portal MSC Infusion, n = 5 Median (IQR)	Arterial MSC Infusion, n = 6 Median (IQR)	*p*-Value *
Weight (kg)	20.5 (20.0–24.7)	23.7 (21.0–24.1)	26.9 (24.6–31.2)	0.061
HR (bpm)	100.0 (71.5–138.0)	97.0 (79.5–102.5)	81.5 (72.3–113.1)	0.835
MABP (mmHg)	61.0 (51.0–74.0)	88.0 (68.0–97.1)	97.8 (71.4–110.0)	0.068
MPABP (mmHg)	9.4 (9.4–9.4)	16.1 (11.3–20.8)	16.7 (8.5–26.8)	0.644
SpO_2_ (%)	100.0 (90.0–100.0)	98.4 (92.9–99.6)	98.4 (95.3–99.0)	0.867
etCO_2_ (mmHg)	4.3 (3.1–5.5)	5.5 (5.0–6.2)	5.0 (4.7–5.8)	0.268
Temperature (°C)	37.9 (36.8–38.5)	38.8 (38.0–39.5)	38.0 (37.4–38.4)	0.159

## Data Availability

Data are available after communication with the corresponding author.

## Supplementary Materials

The following supporting information can be downloaded at https://www.mdpi.com/article/10.3390/cells14191496/s1: Table S1: Primer sequences used in quantitative polymerase chain (qPCR) reaction to track human BM-MSCs in pig tissues (liver, spleen, lung). Figure S1: Hallmark pathway analysis demonstrating no enrichment of hallmark gene sets in differentially expressed genes (DEGs) between samples at baseline and at 6h IRI. Figure S2: No significant differences in concentrations of hepatic and biliary damage markers were observed between the experimental groups during follow-up. Figure S3: Characterization and counts of BM-MSCs before infusion. Figure S4: A schematic representation of the gating strategy used in this study for flow cytometric analysis of MSC expression markers following harvesting. Figure S5: A schematic representation of the gating strategy used in this study for flow cytometric analyses of presence of BM-MSCs in the pulmonary circulation.

## Abbreviations

The following abbreviations are used in this manuscript:

AK5	Adenylate kinase 5
ALP	Akaline phosphatase
ALT	Alanine transaminase
APC	Allophycocyanin
AST	Aspartate transaminase
B2M	Beta-2-microglobulin
BM	Bone marrow
BM-MSC	Bone marrow-derived MSC
CCL26	C-C motif chemokine ligand 26
CT	Cycle threshold
CXCR4	C-X-C chemokine receptor type 4
CYP4F8	Cytochrome P450 family 4 subfamily F member 8
CYP7A1	Cytochrome P450 family 7 subfamily A member 1
DEG	Differentially expressed gene
EPO	Erythropoietin
ES	Enrichment score
FDR	False discovery rate
FITC	Fluorescein isothiocyanate
GAPDH	Glyceraldehyde-3-phosphate dehydrogenase
GGT	Gamma-glutamyl transferase
GZMB	Granzyme B
HE	Hematoxyline and eosine
IQR	Interquartile range
IRI	Ischemia-reperfusion injury
LLB	Left lung base
LLT	Left lung top
MSC	Mesenchymal stromal cell
MVD	Mevalonate diphosphate decarboxylase
MPAP	Mean pulmonary artery pressure
ND4L	NADH-ubiquinone oxidoreductase chain 4L
PAP	Pulmonary artery pressure
PCA	Principal component analysis
PE	Phycoerythrin
PPARA	Peroxisome proliferator-activated receptor alpha
RETN	Resistin
RLB	Right lung base
RLT	Right lung top
RT-qPCR	Reverse transcription-quantitative polymerase chain reaction
SCN9A	Sodium voltage-gated channel alpha subunit 9
SD	Standard deviation
SNORD21	Small nucleolar RNA, C/D box 21
SNORD24	Small nucleolar RNA, C/D box 24
