# Supplementary material for: Optimizing the Infusion Route of Human Bone Marrow Mesenchymal Stromal Cells to Mitigate Liver Ischemia–Reperfusion Injury in a Porcine Model
Source: Cells. 2025 Sep 24;14(19):1496. doi: 10.3390/cells14191496 (PMC12524216; doi:10.3390/cells14191496)
Supplement: Supplementary file 1 [file cells-14-01496-s001.zip › cells-3778586-supplementary.pdf]

Supplementary Tables

Table S1. Primer sequences used in quantitative polymerase chain (qPCR) reaction to track human BM-MSC in pig tissues (liver, spleen, lung).

Gene	Forward Sequence 5′ to 3′	Reverse Sequence 5′ to 3′
Human B2M	GTGTCTGGGTTTCATCCATC	GGCAGGCATACTCATCTTTT
Porcine GAPDH	AAGGTCGGAGTCAACGGATTT	ACCAGAGTTAAAAGCAGCCCTG

Supplementary Figures

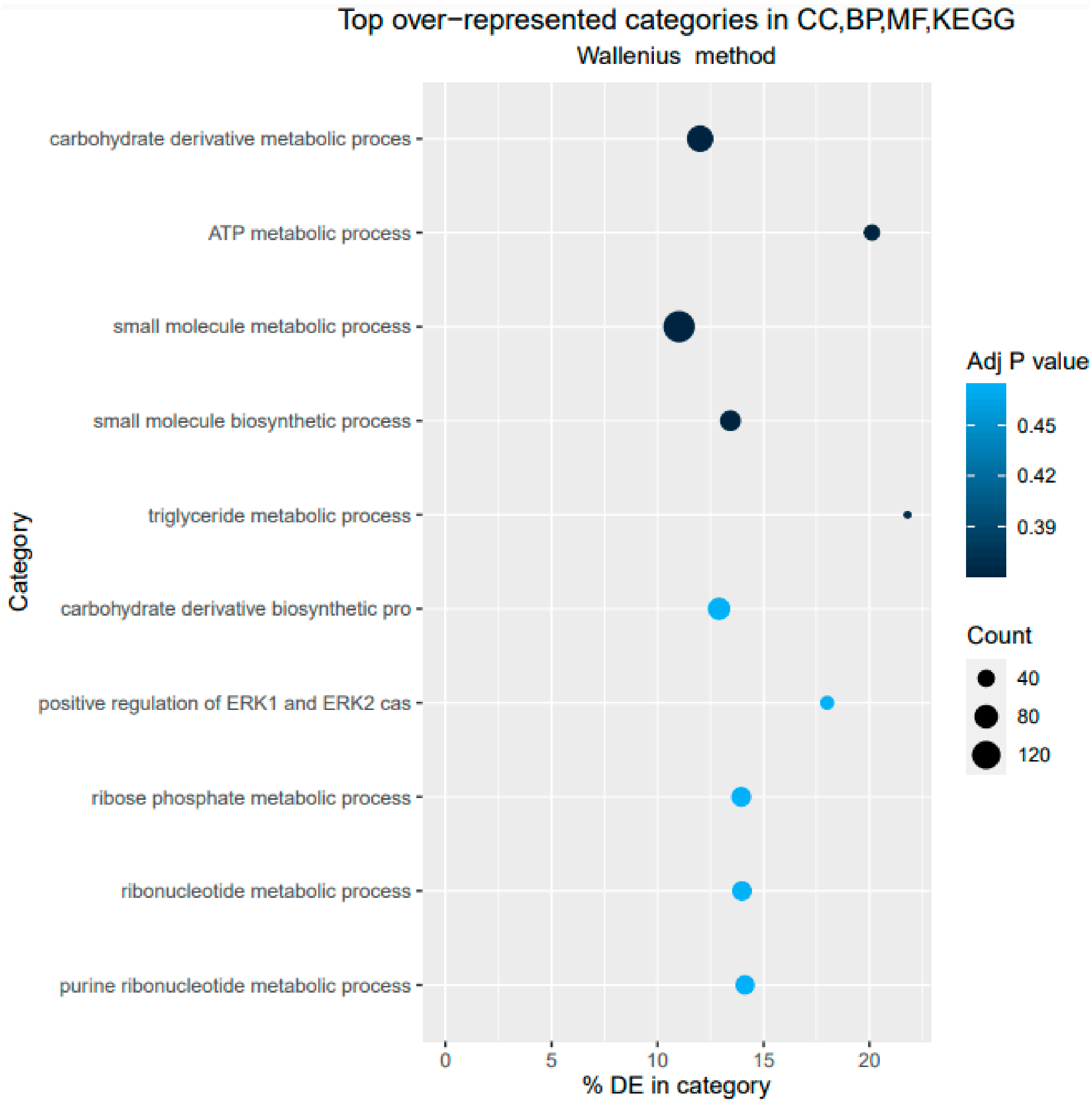

Figure S1. Hallmark pathway analysis demonstrating no enrichment of hallmark gene sets in differentially expressed genes (DEGs) between samples at baseline and at 6h IRI. The DEGs identified by bulk RNA-sequencing were evaluated for functional enrichment using multiple gene annotation databases. GO enrichment analysis was performed and the gene sets were classified according to cell component (CC), biological process (BP), and molecular function (MF). Pathways are orientated along the y-axis and the percentage of differential expression per category is displayed on the x-axis. No significantly downregulated or upregulated gene sets were found between samples at baseline and at 6h IRI.

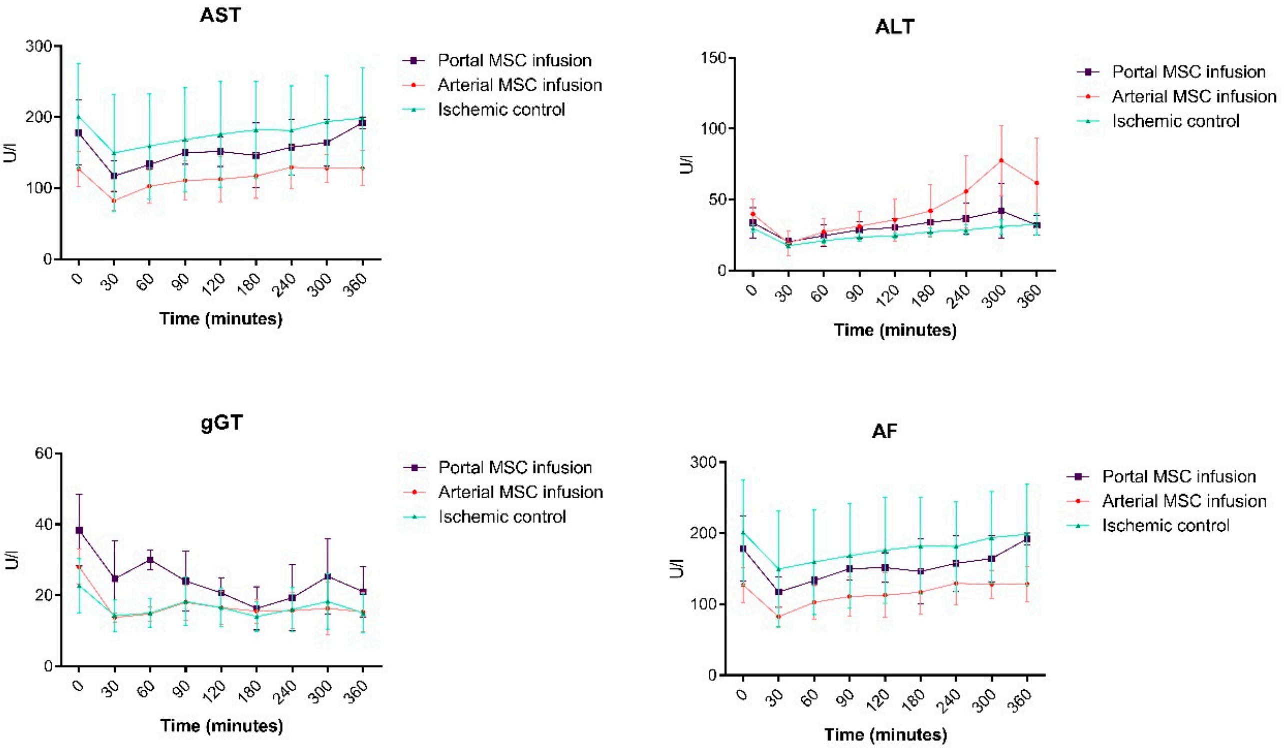

Figure S2. No significant differences in concentrations of hepatic and biliary damage markers were observed between the experimental groups during follow up. Abbreviations: ALP, alkaline phosphatase; ALT, alanine transaminase; AST, aspartate aminotransferase; transaminase; GGT, gamma glutamyl transferase; U/L, units per liter.

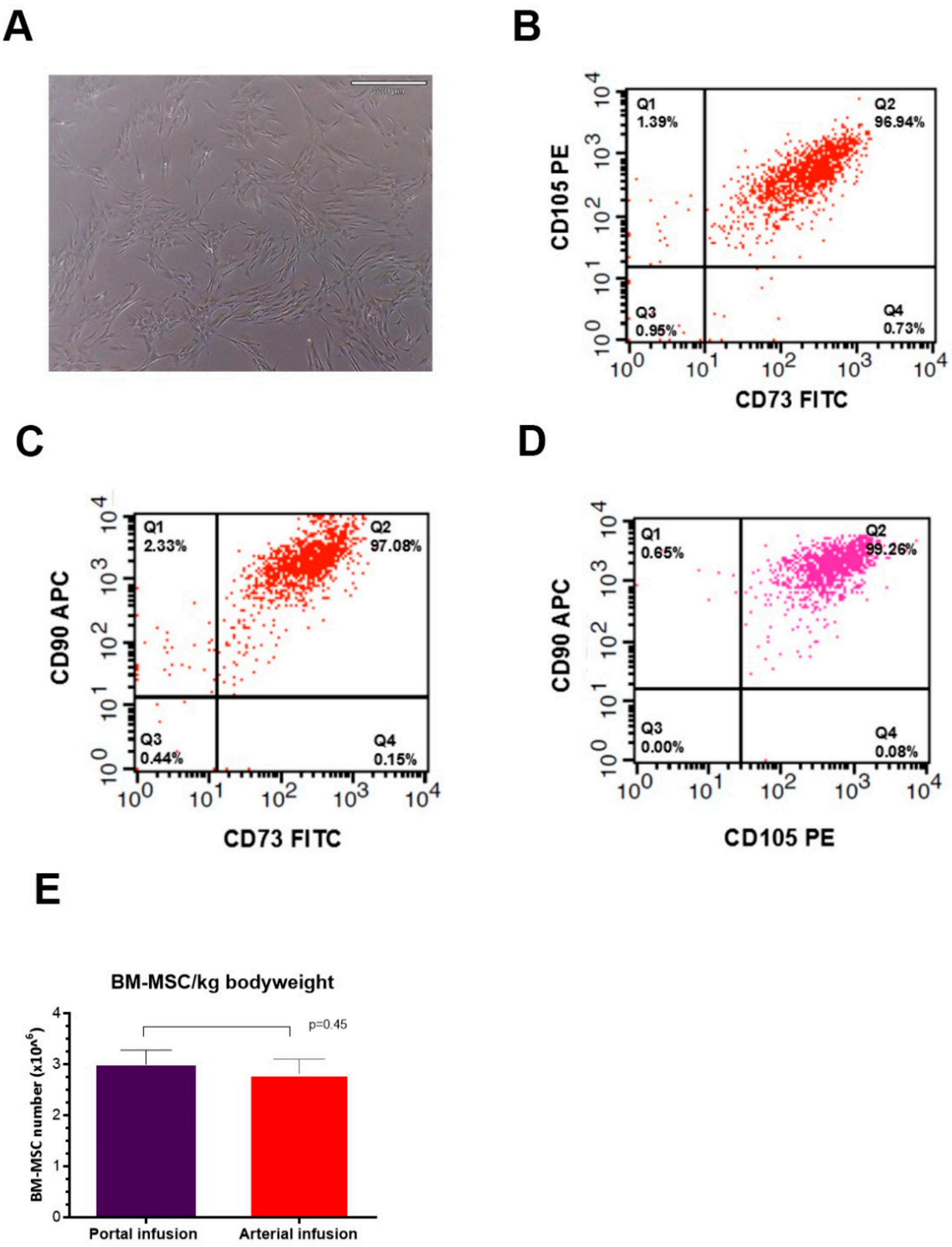

Figure S3. Characterization and counts of BM-MSC before infusion. A, Typical spindle-shape morphology of *in vitro* expanded BM-MSC. Scale bar indicates 680 µm. B,C,D Flow cytometry analysis showing positive surface expression of human CD73, CD90, CD105 in BM-MSC. Representative gating strategy is displayed in Figure S4. F, No significant difference in injected BM-MSC numbers per kilogram bodyweight was found between the portal and arterial group (p=0.45). Abbreviations: APC, allophycocyanin; BM-MSC bone-marrow derived mesenchymal stromal cells; FITC, fluorescein isothiocyanate; PE, phycoerythrin.

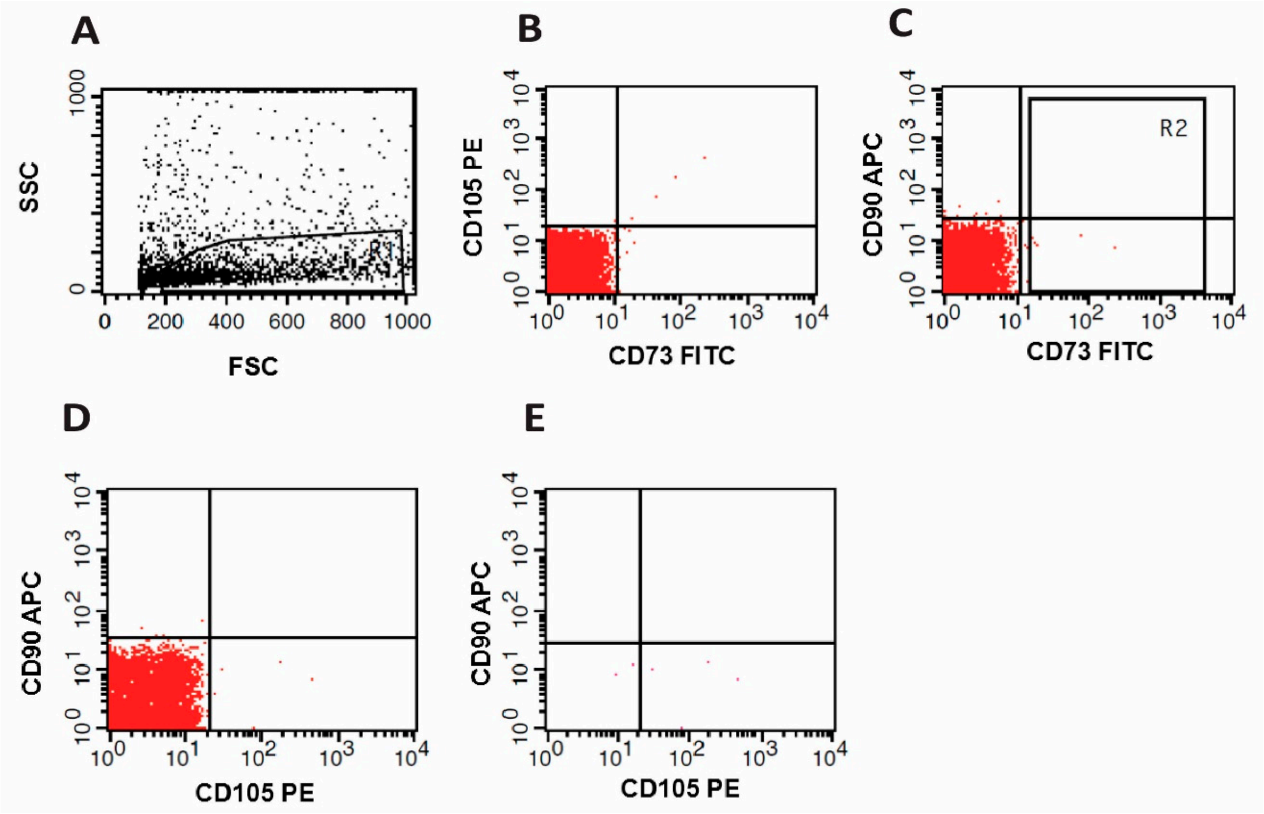

Figure S4. Schematic representative of the gating strategy used in this study for flow cytometric analysis of MSC expression markers following harvesting. Unstained BM-MSC were used as gating controls. A, In the initial step, gate R1 was set to remove debris and dead cells based on scatter characteristics. B-E, quadrant gates were applied to define and locate the negative populations in the left lower quadrant for the used antibodies (CD73 FITC, CD90 APC, CD105 PE). Data were analyzed using FlowJo software. Gating strategy is representative for all samples.

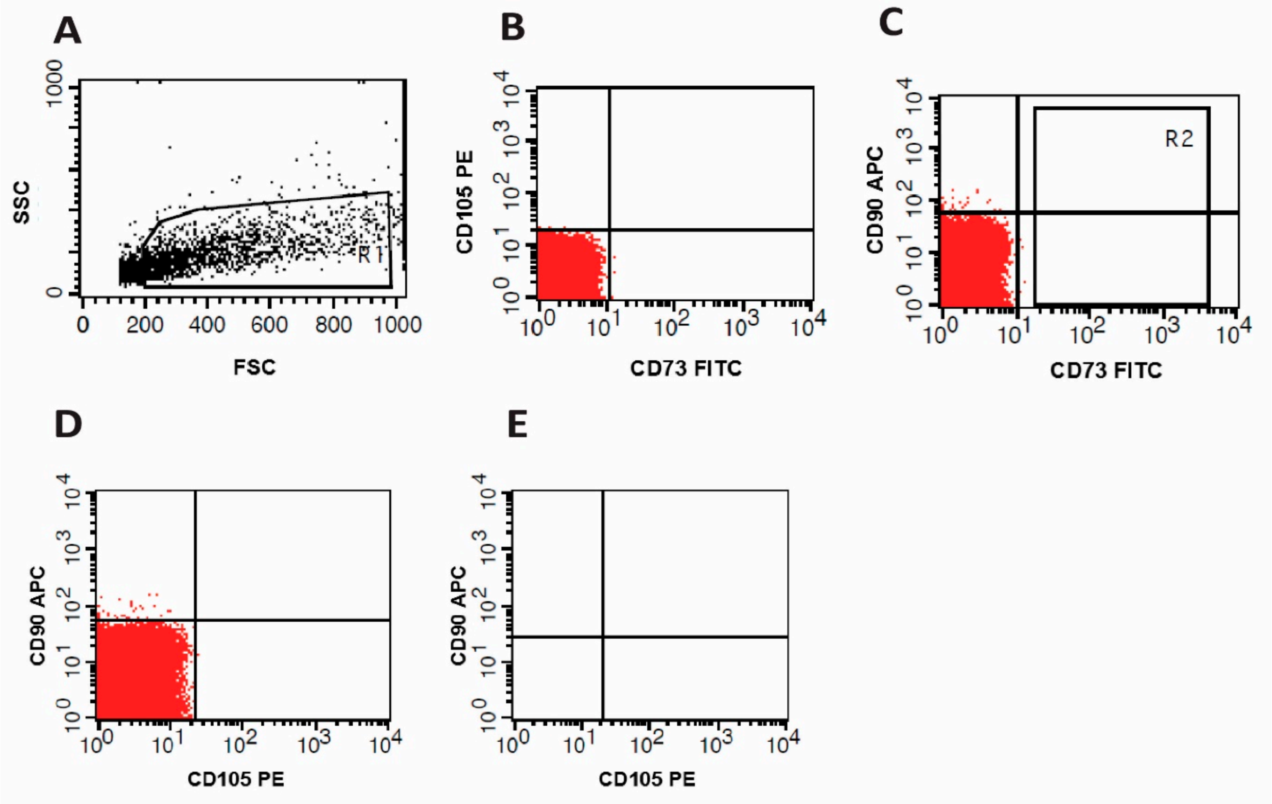

Figure S5. Schematic representative of the gating strategy used in this study for flow cytometric analyses of presence of BM-MSC in the pulmonary circulation. Pulmonary artery blood samples prior BM-MSC infusion were used as gating controls. A, Gate R1 was set to remove debris and dead cells based on scatter characteristics. B-E, quadrant gates were applied to define and locate the negative populations in the left lower quadrant for the used antibodies (CD73 FITC, CD90 APC, CD105 PE). Data were analyzed using FlowJo software. Gating strategy is representative for all pulmonary artery samples.
